# Case Report: Quantitative multimodal imaging for surgical planning in isolated pulmonary artery sling

**DOI:** 10.3389/fped.2025.1689213

**Published:** 2026-01-14

**Authors:** Tianhe Ye, Cong Liu

**Affiliations:** 1 Department of Ultrasound Medicine, Union Hospital, Tongji Medical College, Huazhong University of Science and Technology, Wuhan, China; 2 Hubei Province Key Laboratory of Molecular Imaging, Wuhan, China

**Keywords:** case report, computed tomography angiography (CTA), echocardiography, pediatric, pulmonary artery sling (PAS), quantitative imaging, vascular ring

## Abstract

**Background:**

Pulmonary artery sling (PAS) is a rare congenital vascular anomaly in which the left pulmonary artery (LPA) originates from the right pulmonary artery (RPA), forming a ring around the tracheobronchial tree. Due to non-specific respiratory symptoms, it is frequently misdiagnosed, leading to significant delays in diagnosis. This report emphasizes the crucial role of quantitative multimodal imaging in establishing a definitive diagnosis, stratifying risk, and guiding optimal surgical planning.

**Case presentation:**

A 4-year-and-7-month-old boy presented with a 4-year history of recurrent cough and wheezing that was refractory to standard medical therapy. Echocardiography revealed a dilated main pulmonary artery (MPA) measuring 1.9 cm (*Z*-score +3.2) and an anomalous origin of the LPA from the RPA, with an elevated peak flow velocity of 1.75 m/s. Contrast-enhanced computed tomography angiography (CTA) subsequently confirmed a Type I PAS, enabling precise quantification of a severe focal stenosis of the LPA (minimal diameter 2.8 mm) and demonstrating the absence of significant intrinsic tracheobronchial stenosis. Based on these findings, the patient underwent successful reimplantation of the LPA onto the MPA with an autologous pericardial patch. The postoperative course was uneventful, with complete resolution of respiratory symptoms, and follow-up imaging confirmed a patent anastomosis with successful hemodynamic outcomes.

**Conclusions:**

This case of isolated PAS underscores the indispensable role of a multimodal imaging strategy. While echocardiography can provide initial clues, quantitative CTA is paramount for definitive anatomical classification, precise stenosis quantification, and comprehensive preoperative planning. Early consideration of PAS in children presenting with refractory respiratory symptoms, coupled with advanced imaging, can prevent misdiagnosis and optimize outcomes.

## Introduction

Pulmonary artery sling (PAS) is an uncommon congenital vascular anomaly characterized by an aberrant origin of the left pulmonary artery (LPA) from the posterior aspect of the right pulmonary artery (RPA). The LPA then courses between the trachea and the esophagus to reach the left lung hilum (1, 2). This anatomical configuration forms a vascular ring that frequently compresses the distal trachea or the right mainstem bronchus, leading to significant respiratory distress, such as stridor, biphasic wheezing, and recurrent pulmonary infections (3, 4). Anatomically, PAS is classified into two main types based on the tracheobronchial branching pattern; the present case represents a Type I anomaly, which is characterized by a normal T-shaped carina (5).

Herein, we report a case of isolated PAS in a child who experienced a 4-year diagnostic delay due to refractory respiratory symptoms, consistent with the CARE guidelines (6) (see Supplementary Table S1). Although the clinical utility of multimodal imaging in the diagnosis of PAS is well-established, this report underscores the indispensable role of quantitative multimodal imaging in confirming the diagnosis, guiding successful surgical correction, and establishing PAS as a key differential diagnosis in children with persistent respiratory symptoms unresponsive to conventional therapy. This case illustrates how precise anatomical and hemodynamic data facilitate timely and effective intervention, thereby preventing long-term morbidity associated with delayed diagnosis.

## Case presentation

### Patient information and clinical history

A 4-year-and-7-month-old boy was referred to our institution for evaluation of a persistent cough and expiratory wheezing that had been ongoing for over 4 years and was exacerbated by physical activity. He had been previously treated at various hospitals for presumed bronchopneumonia and refractory asthma, receiving multiple courses of antibiotics, nebulized bronchodilators, and inhaled corticosteroids, which provided only transient symptomatic relief. During the preceding 4 years, he underwent multiple chest X-rays at local clinics, which were repeatedly interpreted as showing non-specific signs suggestive of bronchitis. Review of the maternal history confirmed that routine prenatal ultrasound screening had been performed, with no cardiac or major vascular anomalies reported. The family history was non-contributory for congenital heart or lung disease.

### Clinical findings

Upon admission, the vital signs of the patient were stable. He was well nourished and exhibited normal growth and development, with no evidence of cyanosis. Physical examination revealed diffusely coarse breath sounds bilaterally with expiratory wheezes on chest auscultation. Cardiac examination revealed a regular rhythm with normal S1 and S2 heart sounds and no audible murmurs. No peripheral edema was observed.

### Diagnostic assessment

#### Echocardiography

**Figure 1:**
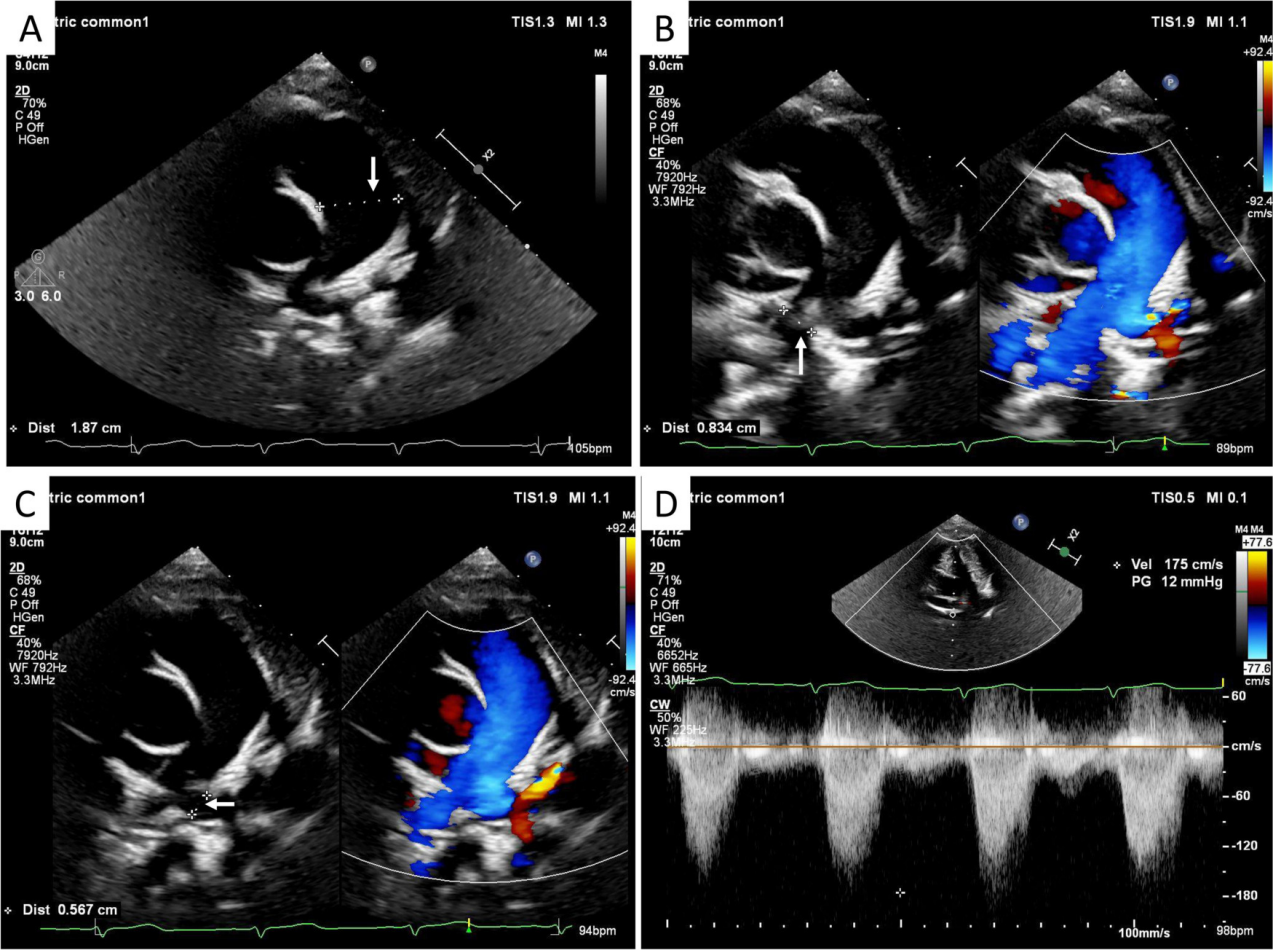
TTE findings. **(A)** Parasternal short-axis view showing marked dilation of the MPA (arrow) to a diameter of 1.9 cm (*Z*-score +3.2). **(B)** High parasternal short-axis view demonstrating the direct continuation of the MPA into the RPA (arrow). **(C)** Suprasternal view illustrating the anomalous origin of the LPA (arrow) from the RPA. **(D)** Continuous-wave Doppler interrogation of the LPA revealing turbulent flow and an elevated peak velocity of 1.75 m/s (peak gradient 12 mmHg), indicating significant stenosis.

Transthoracic echocardiography (TTE) was performed as the initial diagnostic evaluation. It revealed a markedly dilated main pulmonary artery (MPA), measuring 1.9 cm (*Z*-score +3.2 for body surface area) (Figure 1A). The MPA was observed to continue directly into the RPA (Figure 1B). The LPA was identified originating anomalously from the RPA and coursing posterior to the airway structures (Figure 1C). The proximal diameter of the LPA was approximately 0.57 cm, compared to 0.83 cm for the RPA. Continuous-wave and color Doppler flow imaging (CDFI) demonstrated turbulent flow within the LPA with an elevated peak velocity of 1.75 m/s (peak gradient ∼12 mmHg), suggestive of significant stenosis (Figure 1D). No intracardiac shunts or other cardiac anomalies were detected, and all four pulmonary veins drained normally into the left atrium.

#### Computed tomography angiography

**Figure 2:**
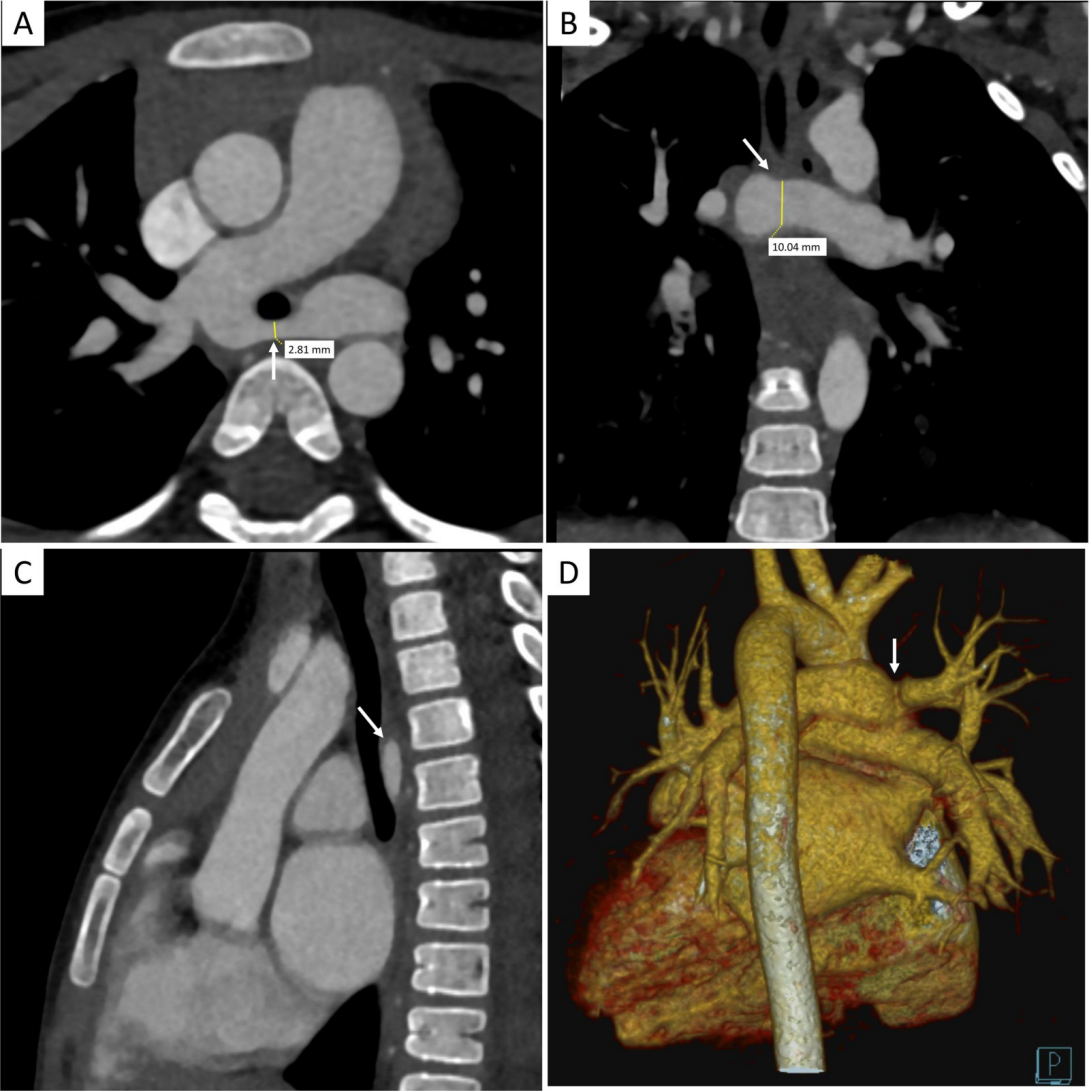
Preoperative multiplanar contrast-enhanced computed tomography angiography (CTA) showing LPA stenosis. **(A)** Axial view demonstrating the severe focal stenosis at the LPA origin, with a minimal anteroposterior diameter of 2.8 mm (yellow line). **(B)** Coronal reformatted view showing the wider craniocaudal dimension of 10.0 mm at the same level (yellow line), revealing the slit-like nature of the stenosis. **(C)** Sagittal view showing the LPA (arrow) coursing between the trachea and esophagus. **(D)** 3D VR reconstruction highlighting the LPA origin (arrow) and its relationship with the great vessels.

**Figure 3:**
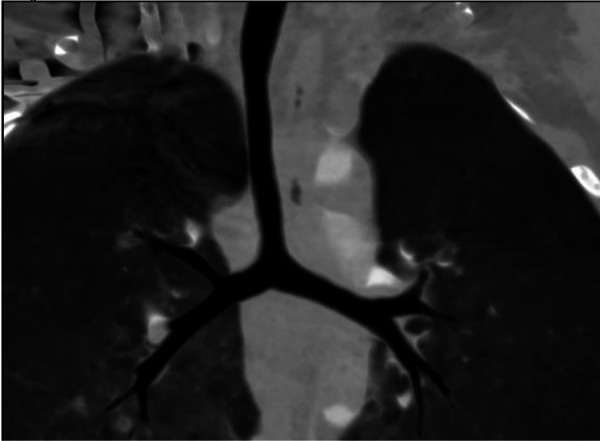
Preoperative coronal CTA image of the airway. The image illustrates the entire airway from the subglottic region to the tracheal bifurcation, showing a normal T-shaped carina and confirming the absence of complete tracheal rings or other intrinsic airway anomalies.

To definitively delineate the complex vascular anatomy and evaluate the tracheobronchial tree, contrast-enhanced, electrocardiogram-gated computed tomography angiography (CTA) was performed, with a CTDIvol of 2.1 mGy and a dose length product (DLP) of 45 mGy·cm. The CTA results confirmed the TTE findings, demonstrating an MPA diameter of 1.9 cm and an RPA diameter of 1.5 cm. The LPA was confirmed to arise from the RPA and to course posteriorly between the trachea and esophagus. Multiplanar reformatted images provided detailed morphological characterization of the stenosis. Critically, axial view confirmed a severe focal stenosis at the origin of the LPA, with a minimal luminal anteroposterior diameter of 2.8 mm, while the coronal view revealed a relatively wider craniocaudal dimension of 10.0 mm (Figures 2A,B). This slit-like morphology of the stenosis explains the moderate Doppler gradient observed on echocardiography despite significant anatomical narrowing. In addition, robust post-stenotic dilation of the LPA to 1.2 cm was evident. Three-dimensional (3D) volume-rendered (VR) and multiplanar reconstructions provided an excellent anatomical roadmap, clearly showing the vascular ring encircling the airway (Figures 2C,D). Importantly, CTA confirmed the absence of “ring–sling complex” anomalies, such as complete tracheal rings or significant bronchomalacia; the trachea and main bronchi were patent and demonstrated normal morphology (Figure 3). Quantitative analysis of the airway revealed a minimum mid-tracheal anteroposterior diameter of 5.8 mm and a transverse diameter of 6.5 mm, which corresponded to approximately 95% of the expected diameter for the patient's age, thereby confirming the absence of significant fixed stenosis (7). Although preoperative bronchoscopy was considered, the high-quality CTA findings demonstrating the absence of complete tracheal rings or fixed stenosis supported the clinical decision to proceed directly with surgical correction to avoid an additional invasive procedure.

### Therapeutic intervention and outcome

Given the definitive diagnosis of isolated PAS with significant anatomical stenosis and associated hemodynamic disturbance, surgical correction was recommended. The patient underwent successful surgical reimplantation of the LPA. Intraoperative findings precisely matched the preoperative imaging results. The surgical procedure involved median sternotomy, establishment of cardiopulmonary bypass, transection of the LPA from its anomalous origin on the RPA, and closure of the RPA stump. An incision was then made in the MPA, and the LPA was anastomosed to the MPA in an anterior, anatomically correct position. The rationale for augmenting the anastomosis with an autologous pericardial patch was to create a wide, tension-free connection, thereby ensuring long-term luminal patency and minimizing the risk of recurrent stenosis. Concurrent tracheoplasty was deemed unnecessary, as preoperative CTA findings confirmed the absence of complete tracheal rings or significant intrinsic stenosis.

**Figure 4:**
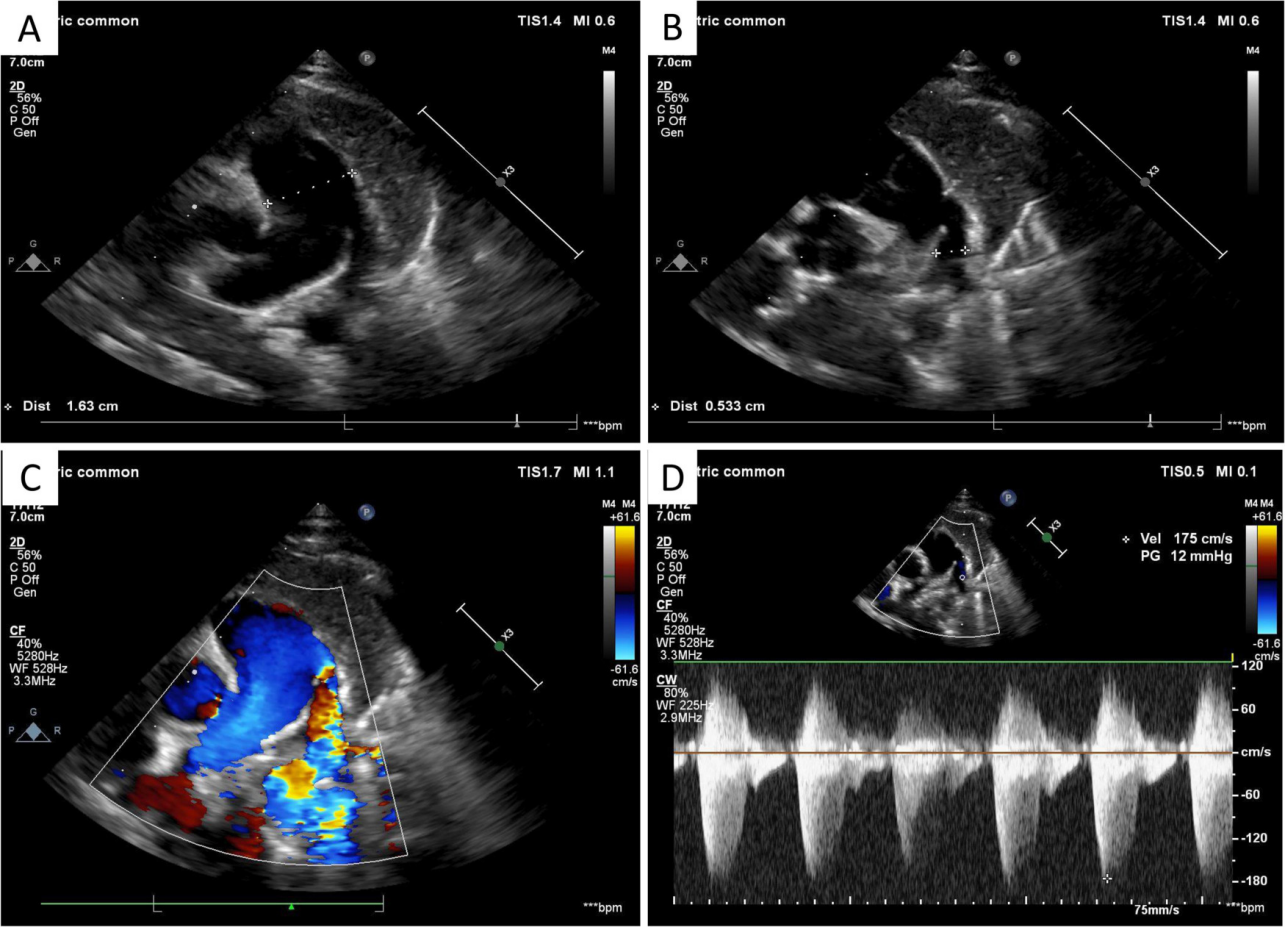
Postoperative bedside TTE. **(A)** Parasternal short-axis view showing the MPA, which measured 1.63 cm in diameter, indicating a reduction from the preoperative size. **(B)** 2D echocardiography view of the reimplanted LPA (arrow) originating from the side wall of the MPA, with a patent proximal diameter of 0.533 cm. **(C)** CDFI demonstrating patent blood flow through the LPA anastomosis with appropriate color filling. **(D)** Continuous-wave Doppler interrogation showing a peak flow velocity of 1.75 m/s (pressure gradient 12 mmHg) across the LPA anastomosis, consistent with a widely patent surgical connection in the early postoperative period.

The postoperative recovery was uneventful. The respiratory symptoms of the patient resolved completely, and he was discharged 2 weeks after surgery. Postoperative imaging was performed to assess the surgical outcome. Bedside TTE revealed that the reimplanted LPA originated from the side wall of the MPA, with a patent proximal diameter of approximately 0.53 cm (Figures 4A,B). CDFI confirmed unobstructed blood flow through the LPA anastomosis, and spectral Doppler analysis showed a peak velocity of approximately 1.75 m/s (corresponding to a pressure gradient of ∼12 mmHg). While the peak velocity remained similar to preoperative levels, likely due to early postoperative vascular compliance or local edema, the flow pattern was laminar, with no evidence of turbulent jetting typically associated with significant obstruction (Figures 4C,D). No residual shunts were observed at the site of patent foramen ovale closure. In addition, a chest X-ray performed on the second postoperative day showed clear bilateral lung markings, with no signs of pulmonary edema, infection, or pleural effusion (Supplementary Figure S1). The surgical wound healed well, and the patient exhibited clear breath sounds bilaterally with no murmurs.

### Patient perspective

Following the successful surgery and the complete resolution of the chronic respiratory symptoms that had impacted the quality of life of their child for 4 years, the parents expressed immense relief and satisfaction with the outcome. They were grateful for the definitive diagnosis that explained the long history of ineffective treatments and expressed optimism regarding future health of their child.

## Discussion

This case report describes the successful diagnosis and management of a rare case of isolated Type I PAS; however, its primary educational value lies in highlighting the diagnostic challenges posed by a 4-year delay. The patient's presentation with chronic cough and frequent wheezing mimicked more common pediatric conditions like asthma or recurrent bronchitis (1, 4). This clinical overlap is a primary contributor to diagnostic delays and underscores a critical clinical pitfall. The persistence of initial working diagnoses despite the lack of response to standard therapies reflects a cognitive bias, in which common conditions are preferentially considered over rarer but more complex etiologies. This case serves as a critical reminder for clinicians to maintain a high index of suspicion for vascular anomalies in children presenting with refractory respiratory symptoms. Failure to reconsider and broaden the differential diagnosis in the setting of treatment failure can lead to prolonged morbidity, unnecessary exposure to medications, and delayed access to definitive care (8).

According to the classification by Wells et al., this case is categorized as a Type I PAS, which is defined by a normal T-shaped tracheal bifurcation. This type is less commonly associated with severe intrinsic tracheobronchial anomalies, such as complete tracheal rings, compared with Type II PAS, which features a low-lying, inverted T-shaped carina and a bridging bronchus (7, 9). This classification was pivotal for our preoperative risk assessment and surgical planning, allowing for a focused vascular repair without the anticipated need for complex airway reconstruction.

A multimodal imaging approach is the cornerstone of modern diagnosis for PAS. TTE serves as an ideal initial screening tool because it is non-invasive, radiation-free, and can identify key indicators, such as MPA dilation, an unusual “high” origin of the LPA, and abnormal flow patterns (10). In the present case, the significant dilation of the MPA (*Z*-score +3.2) represents an important hemodynamic marker. This condition is likely multifactorial but is primarily attributed to the turbulent jet effect originating from the stenotic LPA. High-velocity flow through the narrowed origin generates significant turbulence that propagates retrogradely into the MPA, increasing hemodynamic stress on the vessel wall and leading to progressive vessel dilation (11). However, TTE has limitations in providing a comprehensive assessment of the tracheobronchial tree (12). CTA with 3D reconstruction serves as the gold standard for PAS, providing superior spatial resolution for precise measurement of vessel diameters and stenotic segments (13). The hemodynamic significance of the LPA stenosis was confirmed by the combination of an elevated peak velocity (1.75 m/s) on Doppler echocardiography and severe anatomical narrowing (2.8 mm minimal diameter) with prominent post-stenotic dilation on CTA. In the low-pressure pediatric pulmonary circulation, a peak velocity of this magnitude, together with a >70% reduction in luminal diameter and clear post-stenotic dilation, provides concordant evidence of significant obstruction warranting surgical intervention (14).

The prolonged history of respiratory symptoms in our patient, despite the absence of high-grade fixed tracheal stenosis on static imaging, warrants further discussion. This phenomenon can be attributed to several factors. First, the vascular ring can cause dynamic airway compression, which is most pronounced during expiration and may not be apparent on static imaging studies; however, it becomes symptomatic during periods of increased respiratory effort (15). Second, severe LPA stenosis generates a high-velocity jet that can lead to increased vascular pulsatility against the posterior tracheal wall, causing chronic irritation and inflammation. This pathophysiology helps explain the persistence and progressive worsening of symptoms with age, a process often misinterpreted as worsening asthma (16). A key limitation of our diagnostic workup, which must be explicitly stated, is the absence of dynamic airway assessment. While static CTA is excellent for defining anatomy, it cannot evaluate functional abnormalities like tracheomalacia or dynamic airway collapse during expiration. Furthermore, the lack of postoperative imaging for publication represents another limitation; although follow-up echocardiography confirmed an excellent hemodynamic outcome, postoperative anatomical imaging would have provided a more complete picture of the surgical correction.

Surgical intervention remains the definitive treatment for symptomatic PAS, with excellent outcomes for isolated PAS repair (17). Surgical LPA reimplantation with pericardial patch angioplasty, as performed in our patient, is a validated method to relieve airway compression and restore normal pulmonary vascular anatomy (18). The uneventful postoperative recovery in our case aligns with the literature, which demonstrates that patients without significant intrinsic tracheobronchial anomalies have the most favorable prognoses.

## Conclusion

This case of isolated Type I pulmonary artery sling highlights the essential role of a synergistic, quantitative multimodal imaging approach in overcoming the diagnostic challenges posed by this rare anomaly. Quantitative metrics derived from echocardiography (e.g., vessel *Z*-scores, flow velocities) and CTA (e.g., precise luminal diameters, tracheal dimensions) are critical for accurate diagnosis, effective risk stratification, and detailed surgical planning. This report serves as a crucial reminder for pediatricians and radiologists to maintain a high index of suspicion for PAS in children presenting with refractory or atypical respiratory symptoms, as early and precise diagnosis is paramount to achieving optimal surgical outcomes.

## Data Availability

The original contributions presented in the study are included in the article/**Supplementary Material**; further inquiries can be directed to the corresponding author.

## Supplementary material

Supplementary Figure S1 Postoperative chest X-ray. Image obtained on the second postoperative day, showing clear lung fields bilaterally with no signs of pulmonary edema, pleural effusion, or infection. The normal lung texture supports the clinical finding of resolved respiratory symptoms.
