# Supplementary material for: Case Report: Quantitative multimodal imaging for surgical planning in isolated pulmonary artery sling
Source: Front Pediatr. 2026 Jan 14;13:1689213. doi: 10.3389/fped.2025.1689213 (PMC12847373; doi:10.3389/fped.2025.1689213)
Supplement: Supplementary file 1 [file Table1.docx]

## Supplementary Table 1. CARE Checklist of information to include when writing a case report

| **Section/Topic** | **Item** | **Description** | **Location in Manuscript** |
| --- | --- | --- | --- |
| **Title** | 1 | The diagnosis or intervention of primary focus followed by the words “case report”. | Title |
| **Key Words** | 2 | 2 to 5 key words that identify diagnoses or interventions in this case report. | Keywords Section |
| **Abstract** | 3a | Introduction: What is unique about this case and what does it add to the scientific literature? | Abstract, Background |
|  | 3b | The main symptoms of the patient. | Abstract, Case Presentation |
|  | 3c | The main diagnoses, therapeutic interventions, and outcomes. | Abstract, Case Presentation & Conclusions |
|  | 3d | Conclusion—What is the main “take-away” lesson from this case? | Abstract, Conclusions |
| **Introduction** | 4 | One or two paragraphs summarizing why this case is unique. | Introduction, Paragraph 2 |
| **Patient Information** | 5a | De-identified patient specific information. | Case Presentation, "Patient Information and Clinical History", Paragraph 1 |
|  | 5b | Primary concerns and symptoms of the patient. | Case Presentation, "Patient Information and Clinical History", Paragraph 1 |
|  | 5c | Medical, family, and social history. | Case Presentation, "Patient Information and Clinical History", Paragraph 1 & 2 |
| **Clinical Findings** | 6 | Describe significant physical examination (PE). | Case Presentation, "Clinical Findings" |
| **Timeline** | 7 | Historical and current information from this episode of care organized as a timeline. | Case Presentation (narrative timeline) |
| **Diagnostic Assessment** | 8a | Diagnostic testing (imaging). | Case Presentation, "Diagnostic Assessment" |
|  | 8b | Diagnostic challenges (e.g., prolonged misdiagnosis). | Introduction, Discussion |
|  | 8c | Diagnosis. | Case Presentation, Discussion |
| **Therapeutic Intervention** | 9a | Types of therapeutic intervention (surgical). | Case Presentation, "Therapeutic Intervention and Outcome" |
|  | 9b | Administration of therapeutic intervention (details of the surgery). | Case Presentation, "Therapeutic Intervention and Outcome", Paragraph 1 |
| **Follow-up and Outcomes** | 10a | Clinician- and patient-assessed outcomes. | Case Presentation, "Therapeutic Intervention and Outcome" & "Patient Perspective" |
|  | 10b | Important follow-up diagnostic and other test results. | Case Presentation, "Therapeutic Intervention and Outcome", Paragraph 2 |
| **Discussion** | 11a | A scientific discussion of the strengths and limitations associated with this case report. | Discussion, Paragraph 4 |
|  | 11b | Discussion of the relevant medical literature. | Discussion (all paragraphs) |
|  | 11c | The scientific rationale for any conclusions. | Discussion (all paragraphs), Conclusion |
| **Patient Perspective** | 12 | The patient/family should share their perspective on the treatment(s) they received. | Case Presentation, "Patient Perspective" |
| **Informed Consent** | 13 | Did the patient give informed consent? | Declarations, "Ethics approval and consent to participate" |
