# Supplementary figures and images for: Case Report: Quantitative multimodal imaging for surgical planning in isolated pulmonary artery sling
Source: Front Pediatr. 2026 Jan 14;13:1689213. doi: 10.3389/fped.2025.1689213 (PMC12847373; doi:10.3389/fped.2025.1689213)

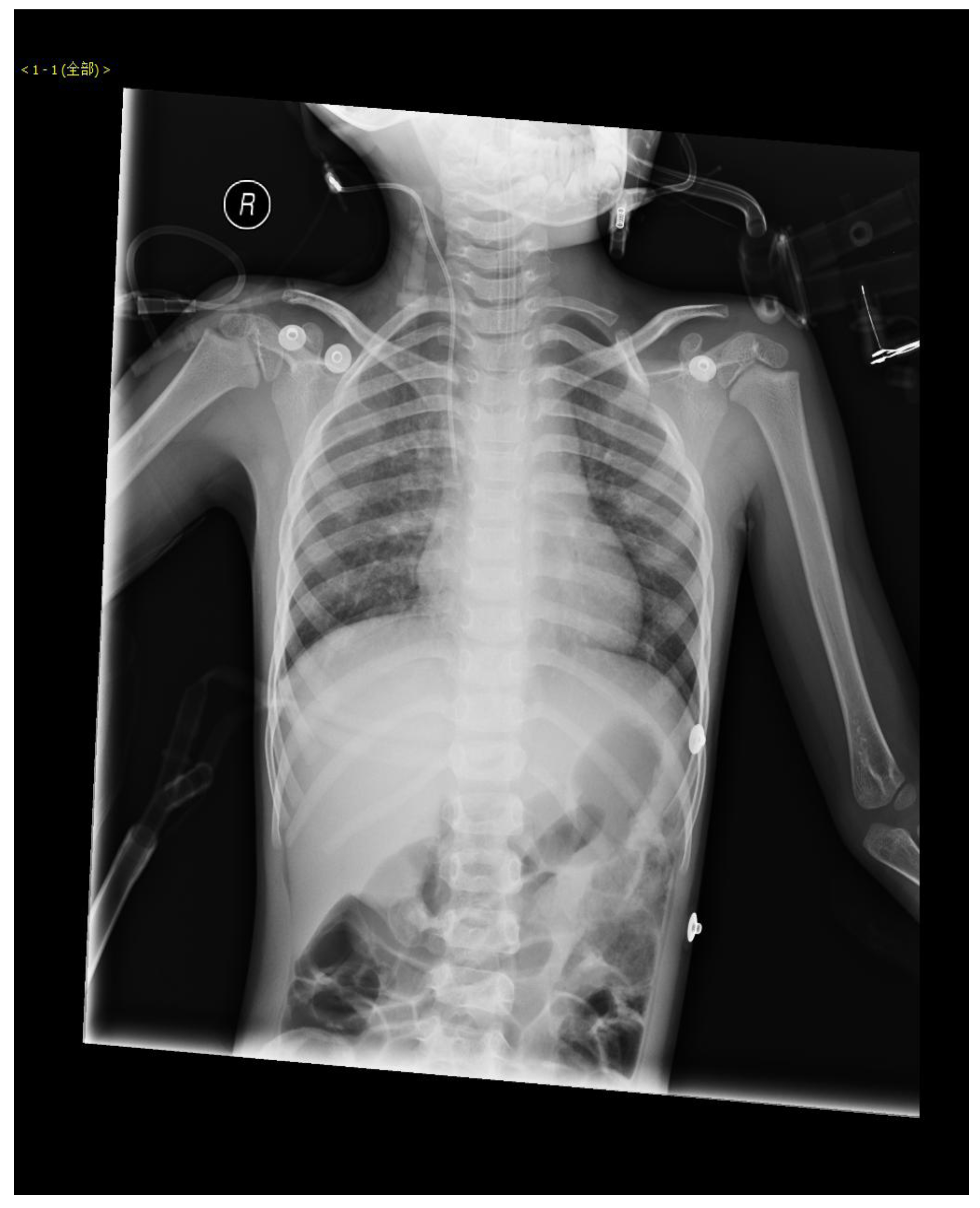

Supplement: Supplementary Figure S1 — Postoperative chest X-ray. Image obtained on the second postoperative day, showing clear lung fields bilaterally with no signs of pulmonary edema, pleural effusion, or infection. The normal lung texture supports the clinical finding of resolved respiratory symptoms. [file Image1.jpeg]
